# The Kids are not alright - When did we start getting more more distressed?

**DOI:** 10.1192/j.eurpsy.2024.422

**Published:** 2024-08-27

**Authors:** N. Glozier, R. Morris, F. Botha, P. Butterworth

**Affiliations:** ^1^University of Sydney, Sydney; ^2^University of Melbourne, Melbourne; ^3^ Australian National University, Canberra, Australia

## Abstract

**Introduction:**

Much has been made of the decline in population mental health over COVID but most studies show this just exacerabted a loing term trend This has predominnatly been attributed to changes in adolescent mental health over the past decade but there ahs been little evalaution of whether this post Millenium cohort was the first to demonstrate such a decline

**Objectives:**

This study investigates to what extent mental differs in people born in different decades – i.e., possible birth cohort differences in the mental health of the popualtion over the past two decades To remove the linear dependency and identify any differences in trends between cohorts, we model mental health for each cohort as a nonlinear smooth function of age in an age-cohort model.

**Methods:**

This analysis draws on 20 annual waves of the Household Income and Labour Dynamic in Australia (HILDA) survey.,is a nationally representative household panel that commenced in 2001 with 13,969 participants. The birth cohort of each person was defined by the decade of birth year(1940s, 1950s, etc). Mental ill health was assessed with the MHI5 from the SF36, in each wave and K10 from alternate waves. We estimate and compare penalized smooth trends in mental health for each cohort using restricted maximum likelihood (REML) using generalized additive mixed modelling (GAMM). Cohort effects are captured by directly estimating the differences between the smooth age trends of adjacent cohorts.

**Results:**

Later cohorts were more likely to have poorer mental health, higher distress, more likely to be single and unemployed, and less likely to be chronically ill or disabled. Mental health was worse for younger age-groups in each survey year, and this discrepancy is much greater in more recent surveys - consistent with a birth cohort effect. Millennials (those born in the early 1990s) had a lower score at the same age as earlier generations, and the later cohorts do not show the age-related improvement seen in other earlier cohorts as they aged. At age 30 the average MHI-5 score of those born in the 1990s was 67, compared to 72.5 and 74 for people born in the 1980s and 1970s.

**Conclusions:**

The deterioration in mental health over time which has been reported in large cross-sectional surveys, likely reflects cohort-specific effects related to the experiences of young people born in the Millennial generation and, to a lesser extent, those from the immediately prior cohort born in the1980s. We need to understand whether later cohorts are less resilient to similar risk factors experienced by earlier cohorts or whether they experience more and/or a greater severity of risks for mental ill-health. Such evidence is critical if the deteriorating pattern of mental health is to be arrested.

**Disclosure of Interest:**

None Declared

